# USING NEURAL NETWORK TO UNCOVER CELL TYPES THROUGH SNRNA SEQUENCING

**DOI:** 10.1093/geroni/igz038.3074

**Published:** 2019-11-08

**Authors:** Fahad Paryani, Vilas Menon

**Affiliations:** Columbia University Vagelos College of Physicians and Surgeons, New York, New York, United States

## Abstract

The advent of single-nucleus RNA-sequencing (snRNAseq) has allowed for the exploration of genetic signatures of the numerous cells in the brain. In particular, snRNAseq data can provide new insights into how many neurodegenerative diseases, such as Alzheimer’s Disease, alter cells in the brain. One major challenge with analyzing snRNAseq data is the lack of a systematic way to classify the various cell types across different datasets. To address this challenge, we developed a general classifier (“DeepSeq”) that uses state-of-the-art deep learning approaches. We trained our model on multiple snRNAseq datasets derived from post-mortem brain tissue in individuals with and without clinical diagnosis of Alzheimer’s Disease from the ROSMAP cohorts. The two snRNAseq datasets contained 70,064 nuclei and 170,275 nuclei. The two studies employed different clustering techniques, and identified 44 and 18 putative cell types. To map these disparate cluster identities across datasets, we extracted the most relevant genes and trained two separate networks, one on each dataset. We then validated each classifier separately on the holdout cells. The resulting classifier accuracy were 87% and 94%. To map clusters across datasets, we then applied each classifier to the other dataset. Both classifiers yielded mappings that reflected the overall biology, correctly categorizing the nuclei into broad and fine cell type classes. Although validation on additional datasets would expand the generality of this approach, our results show that DeepSeq is an easily implementable classification tool that can assign identity to nuclei in new snRNAseq datasets without the need for preprocessing or cross-batch alignment.

